# Associations between type of blood collection, analytical approach, mean haemoglobin and anaemia prevalence in population-based surveys: A systematic review and meta-analysis

**DOI:** 10.7189/jogh.12.04088

**Published:** 2022-11-23

**Authors:** Gretchen A Stevens, Monica C Flores-Urrutia, Lisa M Rogers, Christopher J Paciorek, Fabian Rohner, Sorrel Namaste, James P Wirth

**Affiliations:** 1 Independent Researcher, Los Angeles, California, USA; 2 Department of Epidemiology and Biostatistics, Imperial College London, London, UK; 3 Department of Nutrition and Food Safety, World Health Organization, Geneva, Switzerland; 4 Department of Statistics, University of California, Berkeley, California, USA; 5 GroundWork, Fläsch, Switzerland; 6 The DHS Program, ICF International, Rockville, Maryland, USA

## Abstract

**Background:**

Previous studies have observed that haemoglobin concentrations can be affected by type of blood collection, analysis methods and device, and that near-in-time population-based surveys report substantially different anaemia prevalence. We investigated whether differences in mean haemoglobin or prevalence of anaemia between near-in-time surveys of the same population were associated with differences in type of blood collection or analytic approach to haemoglobin measurement.

**Methods:**

We systematically identified pairs of population-based surveys that measured haemoglobin in the same population of women of reproductive age (WRA) or preschool-aged children (PSC). Surveys were matched on geographic coverage, urban/rural place of residence, inclusion of pregnant women, time of data collection (within 18 months), and, to the extent feasible, age range. Differences in anaemia prevalence were presented graphically. Random-effects meta-analysis and meta-regression of difference in mean haemoglobin were carried out, with subgroups defined by comparison of type of blood collection and analytic approach within each survey pair.

**Results:**

We included 23 survey pairs from 17 countries for PSC and 17 survey pairs from 11 countries for WRA. Meta-regression indicates that surveys measuring haemoglobin with HemoCue® Hb 301 found higher haemoglobin concentrations than near-in-time surveys using HemoCue® Hb 201+ in non-pregnant women ((NPW); 5.8 g/L (95% confidence interval (CI) = 3.2-8.3) mean difference, n = 5 pairs) and PSC (4.3 g/L (1.4-7.2), n = 6). Surveys collecting venous blood found higher haemoglobin concentrations than near-in-time surveys collecting capillary blood in PSC (3.8 g/L (0.8-6.7), n = 8), but not NPW (0.4 g/L (-1.9-2.8), n = 9).

**Conclusions:**

Because this study is observational, differences in haemoglobin concentrations in near-in-time surveys may be caused by other factors associated with choice of analytic approach or type of blood collected. The source or sources of differences should be clarified to improve use of surveys to prioritize and evaluate public health programs.

**Registration:**

PROSPERO CRD42022296553.

Anaemia is a public health problem in many countries, especially among vulnerable groups such as young children and women of reproductive age. During pregnancy, anaemia is associated with increased risk of maternal morbidity, mortality, and low birth weight [1]. Iron deficiency-associated anaemia in women has also been shown to adversely impact work productivity, cognition, and incidence of infection [2]. In children, lower haemoglobin concentrations are associated with delays in cognitive development [3].

In 2012, the World Health Assembly (WRA) endorsed six global nutrition targets as part of the Comprehensive Implementation Plan on Maternal, Infant and Young Child Nutrition. These targets included the reduction, by 2025, of anaemia in women of reproductive age (15-49 years, WRA) by 50% [4]. While progress has been slower than required [5], countries have committed to intensify efforts to decrease all forms of malnutrition, including anaemia, through the UN Decade of Action on Nutrition [6]. Anaemia prevalence in women aged 15 to 49 years is also an indicator (indicator 2.2.3) of the Sustainable Development Goals [7].

The population prevalence of anaemia is often monitored in countries by measurement of haemoglobin concentrations as part of population-based household surveys. The most frequently-conducted representative surveys that contain haemoglobin measurements are surveys collected under The Demographic and Health Survey Program (The DHS Program), which includes DHS surveys and Malaria Indicators Surveys (MIS). Haemoglobin concentrations are also measured in other nutrition and health examination surveys.

There is increasing concern that methodological factors associated with haemoglobin measurement can influence the accuracy and precision of the results, and subsequently affect the estimated prevalence of anaemia [8-10]. Factors that may influence haemoglobin measurements include pre-analytical factors (eg, type of blood collection, lancet type, or capillary blood drop) and the analytical methods and/or device for measuring blood haemoglobin. Errors in haemoglobin measurement could affect assessment of the public health classification of anaemia in countries [11], as well as inference about estimates and trends in anaemia, with important programmatic implications.

There have been numerous studies that examined the difference in the haemoglobin concentration from capillary and venous samples in the same individuals using a consistent analytical approach (ie, chemical method and type of device). Despite some contrary findings [12,13], the majority of studies have found that single drop capillary blood samples yield lower mean haemoglobin concentrations compared to venous blood [8,11,14]. One study found higher haemoglobin in pooled capillary blood than in venous blood [15]. Other studies have compared haemoglobin results from different point-of-care devices frequently used by population-based surveys. Studies suggest that the HemoCue® Hb 301 produces higher haemoglobin concentrations compared to the HemoCue® Hb 201+ device [8,15,16].

Another study design, employed by Hruschka et al. [9] and the Strengthening Partnerships, Results, and Innovations in Nutrition Globally (SPRING) project [10], compared the haemoglobin distribution and anaemia prevalence of near-in-time survey pairs to illustrate how methodological differences in haemoglobin measurement could affect a country’s haemoglobin distribution and overall anaemia prevalence. Both studies found that haemoglobin concentrations were lower in surveys that used capillary blood samples and/or the HemoCue® Hb 201+ device, compared to surveys using venous samples and the HemoCue® Hb 301 device, respectively. However, neither study looked at these pre-analytical and analytical factors separately, and thus could not determine the extent to which each factor may be associated with the differences in haemoglobin observed. Importantly, both studies used data identified by convenience rather than by a systematic approach.

While the aforementioned studies have identified factors that influence the accuracy of haemoglobin measurements, a systematic exploration of the topic has not yet been undertaken. Our analysis takes a systematic approach to comparing haemoglobin and anaemia results from population-based surveys carried out by The DHS Program with other near-in-time population-representative surveys. Our study aimed to identify the extent to which mean haemoglobin concentrations and the prevalence of anaemia in preschool-aged children (PSC), non-pregnant women 15-49 years of age (NPW), and pregnant women (PW) are associated with 1) the type of blood collection (ie, capillary or venous) used, 2) the analytical approach used for measuring haemoglobin (ie, laboratory-based haematological method, HemoCue® device model); and 3) the combined effect of the type of blood collection and analytical method of haemoglobin measurement.

## METHODS

Methods are summarized below, and complete documentation following the Preferred Reporting Items for Systematic Reviews and Meta-Analyses (PRISMA) [17] guidelines is in the **Online Supplementary Document**. We aimed to identify and access data for two populations, PSC and WRA, and do statistical analyses separately for PSC, NPW and PW. However, we retained data on combined groups of WRA if disaggregated data were not available (details below).

### Search strategy

Mean haemoglobin concentrations, anaemia prevalence and relevant data source characteristics (listed in Table S1 in the **Online Supplementary Document**) were obtained on May 27, 2021 from the World Health Organization (WHO) Micronutrients database, part of the Vitamin and Mineral Nutrition Information System (VMNIS) [18]. The VMNIS Micronutrients database is updated via regular searches of 15 bibliographic databases with no language restrictions, data provided by WHO Member States in response to country consultations, and ad hoc contributions of survey reports from WHO’s global network of national, international and academic colleagues (additional details in Appendix S1 in the **Online Supplementary Document**). In addition, we searched The DHS Program website on July 8, 2021, to identify published surveys that included anaemia testing but had not yet been included in the VMNIS Micronutrients database.

### Eligibility criteria

We applied a set of eligibility criteria to the VMNIS Micronutrients database and The DHS Program website in order to create an initial data set for PSC and WRA (details in Appendix S1 in the **Online Supplementary Document**). We considered data from population-based surveys that reported whether data were adjusted for altitude if needed, and that were conducted after 1995 (Appendix S1 in the **Online Supplementary Document**).

### Identification of near-in-time survey pairs

To identify near-in-time pairs of surveys, fieldwork midpoint was computed for each survey by averaging the fieldwork start and end year and month. We retained any survey pairs with a difference in fieldwork midpoint less than or equal to 18 months because 18-month changes in population mean haemoglobin during 2000-2019 were estimated to be less than 1 g/l in all countries and populations (Appendix S1 and Figure S1 in the **Online Supplementary Document**).

### Inclusion of near-in-time survey pairs

From these survey pairs, only pairs including one DHS or MIS survey (hereafter referred to as DHS) and one non-DHS were retained. This approach had the following advantages: 1) the DHS Program is the leading source of population-based haemoglobin data in the highest-anaemia burden countries, and as such, is the de facto control for all comparisons, 2) all data from continuous survey series that share common methods were excluded, and 3) one survey in each pair collected blood by capillary puncture and measured haemoglobin with a HemoCue® device (the DHS) while the non-DHS used a variety of blood collection and analytical methods and/or devices for measuring haemoglobin . In cases where a survey was conducted within 18 months of two other surveys, the closest near-in-time pair was retained to ensure that data from each survey was only utilized for a single near-in-time pair. Finally, we confirmed that both pairs were designed to be representative of the same geographic area (taking into account subnational representativeness of DHS).

### Data harmonization and filling data gaps

For some data sources, including the publicly available surveys from The DHS Program, we obtained the de-identified individual-level microdata with haemoglobin measurements. In cases when the two surveys in a pair did not cover the same age ranges, pregnancy status, or varied in their representativeness (eg, national vs subnational, national urban & rural combined vs national rural only, etc.), microdata were reanalysed to compute summary statistics that were comparable in terms of geographic coverage, urban/rural place of residence, age range, and pregnancy status with those reported for the near-in-time survey. If we obtained mean haemoglobin and/or prevalence of anaemia on all WRA but not on NPW, we included the data provided that we were able to match on inclusion of pregnant women and on definition of anaemia. Finally, we ensured that if needed – both surveys in each pair were adjusted for altitude, but did not ensure that both surveys were adjusted for smoking given the smaller magnitude of the latter adjustment.

In some instances, the non-DHS survey report did not 1) report mean haemoglobin, 2) provide required measures of precision (eg, 95% confidence intervals, standard deviation, standard error, design effects), 3) use a standard haemoglobin cut-off for anaemia [19], 4) specify the type of blood collected, or 5) specify the method of haemoglobin measurement, including model of the HemoCue® device used. To fill data gaps, the research team contacted the principal investigators and requested missing information, and when necessary, for the microdata to be re-analysed (Appendix S1 in the **Online Supplementary Document**). Our selection criteria and data harmonization reduced the risk of bias in our analysis (detailed assessment in Appendix S1 in the **Online Supplementary Document**).

### Effect measures

For each survey pair and demographic group, we computed the following measures: 1) difference in mean haemoglobin concentrations, 2) difference in prevalence of any anaemia (defined as haemoglobin <110 g/L in PSC and PW, and <120 g/L NPW), and 3) difference in prevalence of severe anaemia (defined as haemoglobin <70 g/L in PSC and PW, and <80 g/L NPW). Data and methods for computation of confidence intervals are described in Appendix S1 in the **Online Supplementary Document**. All analyses were done separately for PSC and WRA. If disaggregated data were obtained, data on NPW and PW were analysed separately.

### Classification of survey pairs

As specified in our protocol, survey pairs were classified by similarities or differences in type of blood collection and the analytical approach used for haemoglobin measurement. Since all DHS collected capillary blood, pairs were categorized by the type of blood collected by the non-DHS (capillary or venous). Pairs were also classified by the analytical approach used to measure haemoglobin, non-DHS vs DHS, as follows: laboratory-based vs any HemoCue®, non-DHS HemoCue® model vs DHS HemoCue® model (if they differed), or same/likely same HemoCue® model (details in Appendix S1 in the **Online Supplementary Document**). HemoCue® models were examined separately because the chemical approach used for measuring haemoglobin differed by model (details in Appendix S1 in the **Online Supplementary Document**).

Although we aimed to analyse data on PW separately from data on NPW, we were not able to obtain data disaggregated by physiological status for two surveys, and obtained data on only 8 pairs, with small sample sizes, for PW (**Table 1**). Because around 95% of WRA in the affected countries and years were not pregnant, we grouped the WRA statistics together with the NPW statistics. We present results for PSC and for NPW (17 pairs, including 2 pairs covering WRA; **Table 1**) in the main text, and summary findings for PW in Figure S2 and Figure S4 in the **Online Supplementary Document**.

**Table 1:** Included survey pairs and their characteristics

Geographic coverage	Age range*	Metrics	DHS program survey			Non-DHS program survey				Notes*	Non-DHS program survey reference
**Dates**	**Sample size**	**Analytical approach**	**Dates**	**Sample size**	**Blood collection**	**Analytical approach**
*Non-pregnant women*											
Peru	15-49	TS	Aug 1996-Dec 1996	2123	HemoCue B	1997	772	Venous	HemoCue B	1, 5, 8, 11	Instituto Nacional de Salud, Centro Nacional de Alimentación y Nutritción, Dirección Ejecutiva de Vigilancia Alimentaria y Nutricional. Informe nacional de deficiencia de vitamina A en niños menores de 05 años y mujeres en edad fertil 1997-2001. Lima: Ministerio de Salud; 2001.
Peru	15-49	T	Jul 2000-Nov 2000	5907	HemoCue B	2000	555	Venous	HemoCue B	1, 5, 8, 11	Instituto Nacional de Salud, Centro Nacional de Alimentación y Nutritción, Dirección Ejecutiva de Vigilancia Alimentaria y Nutricional. Informe nacional de deficiencia de vitamina A en niños menores de 05 años y mujeres en edad fertil 1997-2001. Lima: Ministerio de Salud; 2001.
Jordan	15-49	MT	Jul 2002-Sep 2002	2534	HemoCue (not specified)	Oct 2002-Oct 2002	1303	Venous	Automated cell counter (COBAX ABX)	1, 5, 6	Kharabsheh SH, Qarqash Q, Faqih AM.Iron status in preschool Jordanian children of 12-59 mo of age. J Med J. 2006;40:4-13.
9 states, India	20-85	MT	Nov 2005-Aug 2006	23 336	HemoCue Hb 201+	2004-2005	3329	Capillary	Cyanometahaemoglobin	1, 5, 8, 10, 11	Diet and nutritional status of population and prevalence of hypertension among adults in rural areas. National Nutrition Monitoring Board Technical Report No 24. Hyderabad: National Institute of Nutrition, Indian Council of Medical Research; 2006.
Peru	15-49	T	Jan 2004-Sep 2006	5167	HemoCue (not specified)	2004	13 009	Capillary	HemoCue B	1, 5, 8, 11	Monitoreo nacional de indicadores nutricionales 2004. Lima: Ministerio de Salud Publica, Instituto Nacional de Salud; 2004.
Malawi	15-49	MT	Jun 2010-Sep 2010	6473	HemoCue Hb 201+	Jul 2009-Aug 2009	571	Capillary	HemoCue Hb 201+	1, 5	Malawi Government, US Centers for Disease Control and Prevention, UNICEF. A report for the National Micronutrient Survey 2009. Lilongwe: Malawi Government; 2010.
Jordan	15-49	MT	Oct 2009-Dec 2009	6811	HemoCue (not specified)	Mar 2010-Apr 2010	2030	Venous	Beckman Coulter Cell Counter (Beckman Coulter Inc, 2003)	1, 5	Jordan Ministry of Health, Global Alliance for Improved Nutrition (GAIN), United States Center for Disease Control and Prevention (CDC), United Nation Children's Fund (UNICEF, Jordan). National Micronutrient Survey, Jordan 2010. Amman: Jordan Ministry of Health; 2011.
Senegal	15-49	MTS	Oct 2010-Apr 2011	5230	HemoCue Hb 201+	Mar 2010-May 2010	702	Venous	HemoCue Hb 201+	1, 5, 11	Laboratoire de Nutrition - UCAD, Comite Senegalais pour la Fortification des Aliments en Micronutriments (COSFAM), Micronutrient Initiative. Situation de base du statut en vitamine A et en fer chez les enfants de 12-59 mois et chez les femmes en âge de procréer (15-49 ans) dans le cadre du programme de fortification des aliments en micronutriments au Senegal. Dakar: COFSAM; 2011.
Bangladesh	15-49	MT	Jul 2011-Dec 2011	5333	HemoCue Hb 201+	Oct 2011-Dec 2011	1031	Venous	HemoCue Hb 301	1, 5, 6, 11	icddr,b, UNICEF (Bangladesh), GAIN, Institute of Public Health and Nutrition. National micronutrients status survey 2011-12. Dhaka: icddr,b; 2013.
Sierra Leone	15-49	MTS	Jun 2013-Nov 2013	7271	HemoCue Hb 201+	Nov 2013-Dec 2013	871	Venous	HemoCue Hb 201+	1	Ministry of Health and Sanitation (Sierra Leone), UNICEF, Helen Keller International, WHO. Sierra Leone Micronutrient Survey (SLMS). Freetown: Ministry of Health and Sanitation; 2015.
Ethiopia	15-49	MTS	May 2016-Oct 2016	13 462	HemoCue Hb 201+	Mar 2015-Jul 2015	1741	Venous	HemoCue Hb 201+	1, 5, 6	Ethiopian National Micronutrient Survey report. Addis Ababa: Ethiopian Public Health Institute; 2016.
Guatemala	15-49	MT	Oct 2014-Jul 2015	24 117	HemoCue Hb 201+	2015	1512	Capillary	HemoCue Hb 301	1, 5, 6, 8	Informe del Sistema de Vigilancia Epidemiológica de Salud y Nutrición -SIVESNU- 2015, informe final. Guatemala city: Instituto de Nutrición de Centro América y Panamá; 2018.
Malawi	15-49	MTS	Oct 2015-Feb 2016	7374	HemoCue Hb 201+	Dec 2015-Feb 2016	770	Venous	HemoCue Hb 301	2	Malawi Micronutrient Survey 2015-2016
Nepal	15-49	MTS	Mar 2016-Oct 2016	6148	HemoCue Hb 201+	Apr 2016-Jun 2016	2136	Venous	HemoCue Hb 301	2	Nepal 2016 National Micronutrient Status Survey
Tajikistan	15-49	MTS	Aug 2017-Nov 2017	9898	HemoCue Hb 201+	Nov 2016-Nov 2016	2125	Capillary	HemoCue Hb 301	1	Ministry of Health and Social Protection of the Republic of Tajikistan, The World Bank, UNICEF. National Micronutrient Status Survey in Tajikistan, 2016: methodology and tools. Dushanbe: Ministry of Health and Social Protection of the Republic of Tajikistan; 2016.
*Pregnant and non-pregnant women combined*											
Peru	20-49	M	Jan 2004-Sep 2006	5432	HemoCue (not specified)	Aug 2004-Apr 2005	1505	Venous	HemoCue Hb 201+	1, 5, 6, 7	Cárdenas de Jurado HG, Gutiérrez PAM, Arbieto LR, Tasayco FM. Encuesta nacional de indicadores nutricionales, bioquímicos, socioeconómicos y culturales relacionados con las enfermedades crónicas degenerativas. Lima: Ministerio de Salud; 2006.
Ethiopia	15-49	T	Aug 2005-Dec 2005	5899	HemoCue B	Jun 2005-Jul 2005	1135	Capillary	HemoCue Hb 201+	1, 5, 7	Umeta, Haider, Demissie, Akalu, Ayana. Iron Deficiency Anaemia among Women of Reproductive Age in Nine Administrative Regions of Ethiopia. Ethiop.J.Health Dev. 2008;22(3)
*Pregnant women*											
Jordan	15-49	MT	Jul 2002-Sep 2002	330	HemoCue (not specified)	Oct 2002-Oct 2002	108	Venous	Automated cell counter (COBAX ABX)	1, 5, 6	Kharabsheh SH, Qarqash Q, Faqih AM.Iron status in preschool Jordanian children of 12-59 mo of age. J Med J. 2006;40:4-13.
Peru	15-49	T	Jan 2004-Sep 2006	265	HemoCue (not specified)	2004	962	Capillary	HemoCue B	1, 5, 8	Monitoreo nacional de indicadores nutricionales 2004. Lima: Ministerio de Salud Publica, Instituto Nacional de Salud; 2004.
Malawi	15-49	MT	Jun 2010-Sep 2010	677	HemoCue Hb 201+	Jul 2009-Aug 2009	68	Capillary	HemoCue Hb 201+	1, 5	Malawi Government, US Centers for Disease Control and Prevention, UNICEF. A report for the National Micronutrient Survey 2009. Lilongwe: Malawi Government; 2010.
Senegal	15-49	MTS	Oct 2010-Apr 2011	468	HemoCue Hb 201+	Mar 2010-May 2010	99	Venous	HemoCue Hb 201+	1, 5	Laboratoire de Nutrition – UCAD, Comite Senegalais pour la Fortification des Aliments en Micronutriments (COSFAM), Micronutrient Initiative. Situation de base du statut en vitamine A et en fer chez les enfants de 12-59 mois et chez les femmes en âge de procréer (15-49 ans) dans le cadre du programme de fortification des aliments en micronutriments au Senegal. Dakar: COFSAM; 2011.
Sierra Leone	15-49	MTS	Jun 2013-Oct 2013	679	HemoCue Hb 201+	Nov 2013-Dec 2013	174	Capillary	HemoCue Hb 201+	1	Ministry of Health and Sanitation (Sierra Leone), UNICEF, Helen Keller International, WHO. Sierra Leone Micronutrient Survey (SLMS). Freetown: Ministry of Health and Sanitation; 2015.
Guatemala	15-49	MT	Oct 2014-Jul 2015	1385	HemoCue Hb 201+	2015	92	Capillary	HemoCue Hb 301	1, 5, 6, 8	Informe del Sistema de Vigilancia Epidemiológica de Salud y Nutrición – SIVESNU – 2015, informe final. Guatemala city: Instituto de Nutrición de Centro América y Panamá; 2018.
Malawi	15-49	MTS	Oct 2015-Feb 2016	635	HemoCue Hb 201+	Dec 2015-Feb 2016	34	Venous	HemoCue Hb 301	2, 9	Malawi Micronutrient Survey 2015-2016
Nepal	15-43	MTS	Mar 2016-Oct 2016	289	HemoCue Hb 201+	Apr 2016-Jun 2016	204	Venous	HemoCue Hb 301	2	Nepal 2016 National Micronutrient Status Survey
*Children under 5 y of age*											
Peru	6-59	T	Aug 1996-Nov 1996	1128	HemoCue B	1997	495	Venous	HemoCue B	1, 5, 8	Instituto Nacional de Salud, Centro Nacional de Alimentación y Nutritción, Dirección Ejecutiva de Vigilancia Alimentaria y Nutricional. Informe nacional de deficiencia de vitamina A en niños menores de 05 años y mujeres en edad fertil 1997-2001. Lima: Ministerio de Salud; 2001.
Andhra pradesh, India	12-48	MTS	Nov 1998-Dec 1999	866	HemoCue B	Mar 2000-Apr 2000	364	Capillary	HemoCue B	1, 5	State Government of Orissa, WHO, National Institute of Nutrition (India), UNICEF, Micronutrient Initiative. Impact of vitamin A supplementation delivered with oral polio vaccine as part of immunization campaign in Orissa, India (draft final report). Bhubheshwar: State Government of Orissa; 2001.
Orissa, India	12-48	MTS	Nov 1998-Dec 1999	598	HemoCue B	Mar 2000-Apr 2000	323	Capillary	HemoCue B	1, 5	State Government of Orissa, WHO, National Institute of Nutrition (India), UNICEF, Micronutrient Initiative. Impact of vitamin A supplementation delivered with oral polio vaccine as part of immunization campaign in Orissa, India (draft final report). Bhubheshwar: State Government of Orissa; 2001.
Peru	6-59	T	Jul 2000-Nov 2000	2334	HemoCue B	2000	496	Venous	HemoCue B	1, 5, 8	Instituto Nacional de Salud, Centro Nacional de Alimentación y Nutritción, Dirección Ejecutiva de Vigilancia Alimentaria y Nutricional. Informe nacional de deficiencia de vitamina A en niños menores de 05 años y mujeres en edad fertil 1997-2001. Lima: Ministerio de Salud; 2001.
Rural Cambodia	6-59	T	Feb 2000-Jul 2000	1352	HemoCue B	Feb 2000-Sep 2000	1762	Venous	HemoCue B	1, 5	Semba RD, de Pee S, Panagides D, Poly O, Bloem MW. Risk factors for xerophthalmia among mothers and their children and for mother-child pairs with xerophthalmia in Cambodia. Arch Ophthalmol. 2004; 122:517-23.
Jordan	12-59	MT	Jul 2002-Sep 2002	1283	HemoCue (not specified)	Oct 2002-Oct 2002	1060	Venous	Automated cell counter (COBAX ABX)	1, 5, 6	Kharabsheh SH, Qarqash Q, Faqih AM.Iron status in preschool Jordanian children of 12-59 mo of age. J Med J. 2006;40:4-13.
Peru	0-59	T	Jan 2004-Sep 2006	1903	HemoCue (not specified)	2004	12 788	Capillary	HemoCue B	1, 5, 8, 10	Monitoreo nacional de indicadores nutricionales 2004. Lima: Ministerio de Salud Publica, Instituto Nacional de Salud; 2004.
Bolivia	6-23	MT	Feb 2008-Jun 2008	656	HemoCue (not specified)	Nov 2006-Feb 2007	5042	Capillary	HemoCue (not specified)	1, 5	Encuesta Nacional de Nutrición según niveles de vulnerabilidad a la inseguridad alimentaria (línea de base). La Paz: Ministerio de Salud y Deportes; 2007.
Malawi	6-59	MT	Jun 2010-Sep 2010	4475	HemoCue Hb 201+	Jul 2009-Aug 2009	1003	Capillary	HemoCue Hb 201+	1, 5, 9	Malawi Government, US Centers for Disease Control and Prevention, UNICEF. A report for the National Micronutrient Survey 2009. Lilongwe: Malawi Government; 2010.
Jordan	12-59	MTS	Oct 2009-Dec 2009	3383	HemoCue (not specified)	Mar 2010-Apr 2010	902	Venous	Beckman Coulter Cell Counter (Beckman Coulter Inc, 2003)	1, 5	Jordan Ministry of Health, Global Alliance for Improved Nutrition (GAIN), United States Center for Disease Control and Prevention (CDC), United Nation Children's Fund (UNICEF, Jordan). National Micronutrient Survey, Jordan 2010. Amman: Jordan Ministry of Health; 2011.
Senegal	12-59	MTS	Oct 2010-Apr 2011	3452	HemoCue Hb 201+	Mar 2010-May 2010	1486	Venous	HemoCue Hb 201+	1, 5	Laboratoire de Nutrition – UCAD, Comite Senegalais pour la Fortification des Aliments en Micronutriments (COSFAM), Micronutrient Initiative. Situation de base du statut en vitamine A et en fer chez les enfants de 12-59 mois et chez les femmes en âge de procréer (15-49 ans) dans le cadre du programme de fortification des aliments en micronutriments au Senegal. Dakar: COFSAM; 2011.
Bangladesh	6-59	MT	Jul 2011-Dec 2011	2361	HemoCue Hb 201+	Oct 2011-Dec 2011	607	Venous	HemoCue Hb 301	1, 5, 6	icddr,b, UNICEF (Bangladesh), GAIN, Institute of Public Health and Nutrition. National micronutrients status survey 2011-12. Dhaka: icddr,b; 2013.
Kenya	6-59	T	Jul 2010-Sep 2010	3940	HemoCue Hb 301	Sep 2011-Dec 2011	827	Venous	HemoCue Hb 301	3, 4, 5	The Kenya National Micronutrient Survey 2011. Nairobi: Ministry of Health; 2012.
Liberia	6-35	MT	Sep 2011-Dec 2011	1706	HemoCue Hb 201+	Apr 2011-Jun 2011	1445	Venous	HemoCue Hb 201+	1, 4, 5, 6	Liberia Institute of Statistics & Geo-Information Services, UNICEF. Liberia National Micronutrient Survey 2011. Selected preliminary findings, 19 September 2011. Monrovia: Liberia Institute of Statistics & Geo-Information Services; 2011.
Sierra Leone	6-59	MTS	Jun 2013-Nov 2013	5271	HemoCue Hb 201+	Nov 2013-Dec 2013	710	Capillary	HemoCue Hb 201+	1	Ministry of Health and Sanitation (Sierra Leone), UNICEF, Helen Keller International, WHO. Sierra Leone Micronutrient Survey (SLMS). Freetown: Ministry of Health and Sanitation; 2015.
Burkina Faso	6-59	MTS	Sep 2014-Dec 2014	6155	HemoCue Hb 201+	Jun 2014-Jul 2014	2221	Capillary	HemoCue Hb 201+	1, 4	Ministry of Health,Burkina Faso, GroudWork, UNICEF, NI. Enquete Nationale d'Iode et d'Anemie au Burkina Faso 2014 (ENIAB). Ouagadougou:Ministry of Health, Burkina Faso; 2014
Ethiopia	6-59	MTS	May 2016-Oct 2016	8437	HemoCue Hb 201+	Mar 2015-Jul 2015	1375	Venous	HemoCue Hb 201+	1, 5, 6	Ethiopian National Micronutrient Survey report. Addis Ababa: Ethiopian Public Health Institute; 2016.
Guatemala	6-59	MT	Oct 2014-Jul 2015	10 854	HemoCue Hb 201+	2015	686	Capillary	HemoCue Hb 301	1, 5, 6, 8	Informe del Sistema de Vigilancia Epidemiológica de Salud y Nutrición – SIVESNU – 2015, informe final. Guatemala city: Instituto de Nutrición de Centro América y Panamá; 2018.
Malawi	6-59	MTS	Oct 2015-Feb 2016	5227	HemoCue Hb 201+	Dec 2015-Feb 2016	1174	Venous	HemoCue Hb 301	2, 9	Malawi Micronutrient Survey 2015-2016
India	12-59	TS	Jan 2015-Dec 2016	194 349	HemoCue Hb 201+	Feb 2016-Oct 2018	11 237	Venous	Cyanometahaemoglobin	1, 5	Ministry of Health and Family Welfare, Government of India, UNICEF, Population Council. Comprehensive National Nutrition Survey (CNNS) national report. New Delhi: Ministry of Health and Family Welfare; 2019.
Nepal	6-59	MTS	Mar 2016-Oct 2016	2177	HemoCue Hb 201+	Apr 2016-Jun 2016	1651	Venous	HemoCue Hb 301	2	Nepal 2016 National Micronutrient Status Survey
Tajikistan	6-59	MTS	Aug 2017-Nov 2017	5460	HemoCue Hb 201+	Nov 2016-Nov 2016	2097	Capillary	HemoCue Hb 301	1	Ministry of Health and Social Protection of the Republic of Tajikistan, The World Bank, UNICEF. National Micronutrient Status Survey in Tajikistan, 2016: methodology and tools. Dushanbe: Ministry of Health and Social Protection of the Republic of Tajikistan; 2016.
Ghana	6-59	MTS	Oct 2016-Dec 2016	3077	HemoCue Hb 201+	Apr 2017-Jun 2017	1018	Capillary	HemoCue Hb 301	1, 5	University of Ghana, GroundWork, University of Wisconsin-Madison, KEMRI-Wellcome Trust, UNICEF. Ghana Micronutrient Survey 2017. Accra: University of Ghana; 2017.

M – mean, T – prevalence of total anaemia, S – prevalence of severe anaemia, DHS – Demographic and Health Survey

*Years for women, months for children

†Notes:

1 – Individual-level data were reanalysed (DHS survey only).

2 – Individual-level data were reanalysed (DHS and non-DHS survey).

3 – No individual level data were reanalysed

4 – DHS survey is a Malaria Indicator Survey.

5 – Non-DHS survey design effect was not reported, estimated as described in Appendix S1 in the Online Supplementary Document.

6 – Non-DHS survey standard deviation of haemoglobin not reported, estimated as described in Appendix S1 in the Online Supplementary Document.

7 – Non-DHS survey data were not reported by pregnancy status.

8 – Non-DHS survey fieldwork months not known.

9 – Non-DHS survey is a subsample of the DHS survey.

10 – Surveys were not matched on age range.

11 – Non-DHS survey excluded lactating women.

### Statistical analysis

Quantitative meta-analysis of prevalence difference was not appropriate because difference in prevalence is expected to depend on underlying prevalence, which varied substantially in our data set. Therefore, rather than quantitatively synthesizing difference in prevalence of anaemia, the data were displayed graphically against prevalence estimated by the DHS.

Restricted maximum-likelihood random-effects meta-analyses of difference in mean haemoglobin were carried out. We treated the DHS as the control group (since these surveys used capillary blood collection and a HemoCue® device) and the non-DHS to be the comparator group. Pre-specified sub-group analyses were carried out on the basis of type of blood collection and by the analytical approach of haemoglobin measurement as described above. As the aforementioned sub-group analyses may each be confounded by the other factor, we performed two restricted maximum-likelihood random-effects meta-regressions, one for each population group, in an attempt to disentangle any effects associated with these factors. All statistical analyses were carried out using Stata version 16.1.[20].

### Ethical considerations

Following the procedures of WHO’s Ethics Review Committee, this protocol is exempt from ethical review as “Protocols are exempt from review when there is no possibility of harm arising as a result of the conduct of the research project, or if the information being evaluated is already in the public domain.”

## RESULTS

### Survey pairs

We included 23 survey pairs from 17 countries for PSC and 17 survey pairs from 11 countries for WRA (Figure S1 in the **Online Supplementary Document**, **Table 1**, data set available online at https://osf.io/g895r/). All survey pairs were from low- or middle-income countries, and data collection ranged from 1996 to 2017. All included statistics were matched on time of data collection (midpoint within 18 months), geographic coverage, place of residence, anaemia definition (including altitude adjustment), and demographic group (including pregnancy status). We included two survey pairs – India 9 states 2004-2005 (WRA) and Peru 2004 (PSC) – where we were not able to match on age range because DHS covered a narrower age range than the non-DHS, and we were not able to reanalyse the non-DHS (**Table 1**).

The number of survey pairs included varied by analysis because we were not able to obtain mean haemoglobin, prevalence of total anaemia and prevalence of severe anaemia for all surveys. Considering all surveys included in any analysis, more than half of non-DHS used venous blood (11/17 for WRA, 13/23 for PSC). Regarding devices used, the majority of non-DHS (14/17 for WRA, 20/23 for PSC) and all DHS used a HemoCue® device. The non-DHS were more likely to use a HemoCue® Hb 301 than the DHS (5/17 for WRA, 7/23 for PSC vs 0/17 for WRA, 1/23 for PSC). Four non-DHS used laboratory-based methods: the cyanmethemoglobin method was used by India’s 2004-05 survey in WRA and 2016-2018 surveys in PSC, and automated haematology analysers were used by Jordan’s 2010 (Beckman Coulter) and 2002 surveys (COBAX ABX; Table S2 in the **Online Supplementary Document**, **Table 1**). As these surveys were few, methodologically heterogeneous and limited to two countries, comparisons of these survey pairs should be interpreted with caution.

### Anaemia prevalence

**Figure 1:**
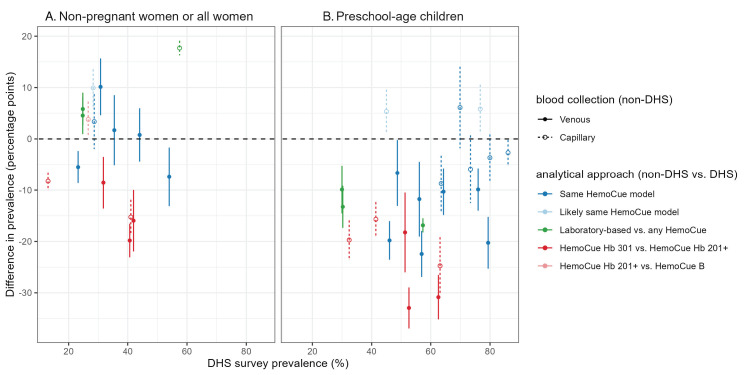
Difference in prevalence of anaemia (percentage points) in near-in-time survey pairs, non-Demographic and Health Survey (DHS) anaemia prevalence *minus* DHS anaemia prevalence. **Panel A.** Non-pregnant women/all women. **Panel B.** preschool-aged children.

Across all surveys and demographic groups, the prevalence of anaemia ranged from 4.8% to 86.1% (median = 41.5%, interquartile range (IQR = 28.6%-56.9%, n = 95). Differences in the anaemia prevalence between 47 near-in-time survey pair/demographic units (ie, non-DHS anaemia prevalence *minus* DHS anaemia prevalence) ranged from -32.9 to 17.7 percentage points (pp), with a median of -8.3 (IQR = -17.6,3.8) pp. We plotted differences in anaemia prevalence against prevalence of anaemia in the DHS because the difference in prevalence is expected to be related to underlying prevalence, with smaller differences in countries with very high or very low prevalence of anaemia (**Figure 1**, Figure S3 in the **Online Supplementary Document**). For all identified survey pairs where the non-DHS used HemoCue® Hb 301 and the DHS used HemoCue® Hb 201+, the estimated prevalence of anaemia was lower in the non-DHS survey regardless of type of blood collection (**Figure 1**, Table S3 in the **Online Supplementary Document**; median difference of -15.3 pp for NPW (n = 5) and -22.2 pp for PSC (n = 6)). In contrast, non-DHS measuring haemoglobin with the same or likely the same HemoCue® reported higher and lower prevalence than their near-in-time DHS (**Figure 1**). For PSC, surveys collecting venous blood also consistently reported lower prevalence of anaemia than the near-in-time DHS that collected capillary blood regardless of analytical approach used (median difference -16.9 pp, IQR = 10.3-20.3 pp; n = 13; Table S3 in the **Online Supplementary Document**). In contrast, non-DHS collecting venous blood in women reported both higher and lower prevalence of anaemia than their near-in-time DHS, with a median difference of -2.4 pp (IQR = -8.5,4.6 pp, n = 10).

The public health significance of anaemia in a population is based on the prevalence of anaemia [21]. In 5 of 16 (NPW) and 9 of 23 (PSC) near-in-time survey pairs, the classification differed between the two surveys in the pair (Figure S2 in the **Online Supplementary Document**). The most-common discordant classification observed (NPW, n = 6; PSC, n = 3) was a DHS Program survey indicating a “severe” public health significance and the corresponding non-DHS survey indicating a “moderate” public health significance.

### Mean haemoglobin

#### Women 15-49 years

Across 30 included surveys covering NPW or all WRA, mean haemoglobin ranged from 107 to 140 g/L (median = 125, IQR = 121-128). The difference between 13 near-in-time survey pairs (non-DHS less DHS) ranged from -8 to 7 g/L, with a median of 0 (IQR = -2,3) g/L.

**Figure 2:**
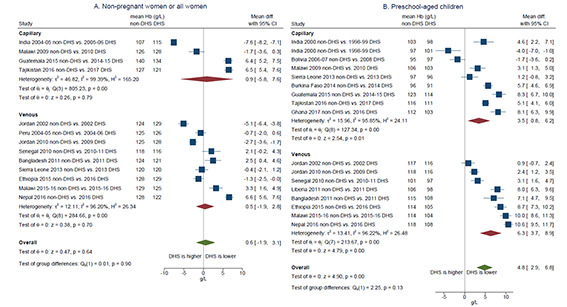
Difference in mean haemoglobin in near-in-time survey pairs, non-Demographic and Health Survey (DHS) anaemia prevalence *minus* DHS anaemia prevalence, grouped by type of blood collected in the non-DHS. **Panel A.** Non-pregnant women/all women. **Panel B.** Preschool-aged children. Notes: 1. Peru 2004-05 non-DHS and 2004-2006 DHS data include pregnant women. All other data are for non-pregnant women. 2. All DHS program surveys collected capillary blood.

**Figure 3:**
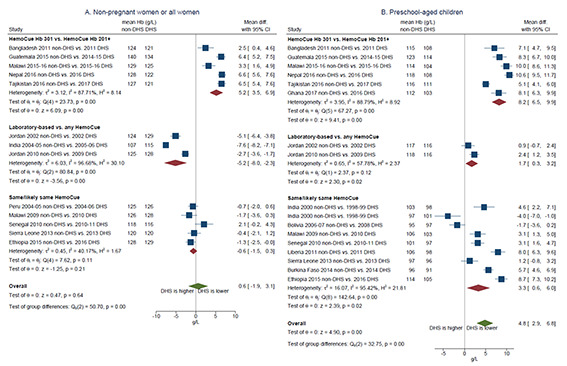
Difference in mean haemoglobin in near-in-time survey pairs, non-Demographic and Health Survey (DHS) anaemia prevalence *minus* DHS anaemia prevalence, grouped by concordance/discordance of analytic approach used to measure haemoglobin concentration. **Panel A.** Non-pregnant women/all women. **Panel B.** Preschool-aged children. Note: Peru 2004-05 non-DHS and 2004-2006 DHS data include pregnant women. All other data are for non-pregnant women.

Random-effects meta-analysis showed that the difference in mean haemoglobin (non-DHS less DHS) was not statistically different from zero when considering all studies pooled together or when grouping by type of blood collection in the non-DHS (**Figure 2**). However, subgroup analysis by analytical approach (**Figure 3**) showed that non-DHS using HemoCue® Hb 301 tend to report higher mean haemoglobin than near-in-time DHS using HemoCue® Hb 201+, with a pooled mean difference of 5.2 (95% CI = 3.5-6.9) g/L (n = 5; *P* < 0.001), while those using the same or likely the same HemoCue® model reported similar mean haemoglobin (pooled mean difference of -0.6 (-1.5,0.3) g/L; *P* = 0.21). As noted above, the small number of non-DHS using laboratory-based methods led to wide confidence intervals in the subgroup analysis (**Figure 3**).

Each of the comparisons above might have been confounded by the other factor, for example, if there are systematic differences associated with type of blood collection and with analytical approach, and the two factors are correlated. Sub-group analysis considering all possible interactions of type of blood collection and analytical approach resulted in 1-4 survey pairs per group (Figure S4 in the **Online Supplementary Document**), but was consistent with the analyses in **Figure 2** and **Figure 3**. Meta-regression was similarly limited by the small number of survey pairs included (n = 13). However, it indicated that, on average, non-DHS using HemoCue® Hb 301 report higher mean haemoglobin (coefficient of 5.8 (3.2-8.3) g/L) than near-in-time DHS using HemoCue® Hb 201+ (**Table 2**). The meta-regression also found no difference between surveys collecting venous or capillary blood (coefficient of 0.4 (-1.9,2.8) g/L). Differences in mean haemoglobin in pregnant women showed similar patterns by type of blood collection and analytical approach as well (Figure S4 in the **Online Supplementary Document**).

**Table 2:** Fitted coefficients from random effects meta-regression of the difference in mean hemoglobin (g/L), non-DHS survey less DHS survey, in each survey pair. Two regressions were fitted, one for non-pregnant women or women pregnancy status was not reported, and one for preschool-aged children. Indicator variables were included when the type of blood collection or analytic approach used by the non-DHS survey differed from that of its near-in-time DHS survey. The intercept corresponds to the difference in mean haemoglobin when both surveys collected blood via capillary puncture and analyzed haemoglobin using the same HemoCue® model (either confirmed or suspected)

Differing survey method (indicator variables)	Number of survey pairs	Fitted regression coefficient and 95% CI (g/L)	P-value
*Non-pregnant women/women (n=13 study pairs)*			
Venipuncture (non-DHS) vs. capillary puncture (DHS)	9	0.4 (-1.9, 2.8)	0.712
HemoCue® Hb 301 (non-DHS) vs. HemoCue® Hb 201+ (DHS)	5	5.8 (3.2, 8.3)	<0.001
Laboratory-based methods (non-DHS) vs. any HemoCue® (DHS)	3	-4.6 (-7.5, -1.8)	0.001
Intercept		-0.8 (-3.5, 1.8)	0.536
*Preschool-aged children (n=17)*			
Venipuncture (non-DHS) vs. capillary puncture (DHS)	8	3.8 (0.8, 6.7)	0.012
HemoCue® Hb 301 (non-DHS) vs. HemoCue® Hb 201+ (DHS)	6	4.3 (1.4, 7.2)	0.004
Laboratory-based methods (non-DHS) vs. any HemoCue® (DHS)	2	-4.2 (-8.8, 0.4)	0.075
Intercept		2.1 (0.0, 4.2)	0.053

#### Preschool-aged children

Across 40 included surveys covering PSC, mean haemoglobin ranged from 91 to 122 g/L (median = 106, IQR = 99-114). Difference between 17 near-in-time survey pairs (non-DHS less DHS) ranged from -4 to 11 g/L, with a median of 5 (IQR = 2-8) g/L.

Pooling the mean differences from 17 included survey pairs by random-effects meta-analysis, non-DHS mean haemoglobin was 4.8 (2.9-6.8) g/L higher than DHS (*P* < 0.001). Single-factor sub-group analysis by type of blood collection in the non-DHS did not explain this difference (**Figure 2**; test of group differences Qb(1) = 2.25, *P* = 0.13). Sub-group analysis by analytic device indicated that the mean difference may be larger when the non-DHS uses the HemoCue® Hb 301 (8.2 (6.5-9.9) g/L, *P* < 0.001) vs when the same HemoCue® model is used (3.3 (0.6-6.0) g/L, *P* = 0.02; test of group differences Qb(1) = 32.75, *P* < 0.001; **Figure 3**).

As with the analysis of mean differences in haemoglobin for NPW, the single-factor comparisons presented in **Figure 2** and **Figure 3** may each be confounded by the other factor. Sub-group analysis considering all possible combinations of type of blood collection and analytic method /device are shown in Figure S4 in the **Online Supplementary Document**. Considering only survey pairs where both surveys collected capillary blood and were confirmed/likely to use the same HemoCue® model for analysis of haemoglobin, the difference in mean haemoglobin (non-DHS less DHS) was not significantly different from zero (1.6 (-1.3,4.6) g/L, *P* = 0.29, n = 6). Other sub-groups were consistent with higher mean haemoglobin measured in the non-DHS, with two to three survey pairs in each group. Assuming that there is no statistical interaction between effects associated with type of blood collection and analytical approach, meta-regression may clarify these effects (**Table 2**). For the comparison of the device for haemoglobin measurement, meta-regression coefficients for PSC were similar to coefficients for NPW: non-DHS using HemoCue® Hb 301 was associated with a mean haemoglobin 4.3 (1.4-7.2) g/L higher than its near-in-time DHS (*P* = 0.004), and results for laboratory-based methods were similar to those for NPW (**Table 2**). However, unlike for NPW, non-DHS collecting venous blood reported mean haemoglobin 3.8 (0.8-6.7) g/L higher than their near-in-time DHS (*P* = 0.012).

## DISCUSSION

Our study compares near-in-time population-representative surveys to determine whether type of blood collection or analytic approach is associated with results of haemoglobin measurements collected under typical field conditions. We found that haemoglobin concentrations were associated with haemoglobin measurement device in both PSC and NPW, with mean haemoglobin concentrations around 4 - 6 g/L higher in non-DHS using the HemoCue® Hb 301 device compared to the near-in-time DHS using HemoCue® Hb 201+. In PSC only, surveys collecting capillary blood reported lower mean haemoglobin concentrations than near-in-time surveys collecting venous blood.

Both in PSC and NPW, the differences in haemoglobin concentrations in near-in-time survey pairs produced sizable differences in anaemia prevalence that, in many instances, altered the “public health significance” classification [19]. The differences in anaemia prevalence and public health classification found in near-in-time survey pairs creates a challenging situation for public health officials and researchers, as the burden of anaemia at the population level is used to set public health priorities [22,23] and guide the choice of public health interventions implemented [24,25].

Our analysis of near-in-time surveys included 4 of 5 survey pairs described in previous studies that compared near-in-time survey pairs identified by convenience [9,10]. Hrushka et al. [9] included a survey pair from Cameroon (2011 DHS and 2009 non-DHS), which we excluded because the fieldwork midpoint difference was greater than 18 months.

### Differences by type of blood collection

Our study found that mean haemoglobin concentrations were higher in non-DHS using venous samples compared to their DHS counterpart that used capillary samples in PSC (by around 4 g/l) but not NPW. Other studies that collected both venous and capillary blood samples from the same children in field settings have found similar results using both the HemoCue® Hb 301 device [11,26] and HemoCue® 201+ [27] devices. However, some studies have found higher haemoglobin levels from capillary samples in children [13,28] using the HemoCue® B device.

The lack of association we found between type of blood collection and mean haemoglobin concentration in NPW is reflected in the literature as there is no consistent bias in haemoglobin concentration associated with capillary vs venous blood in women [14,16,28,29]. Some studies note that capillary drop-to-drop variability in haemoglobin measurement is higher than for pooled capillary or venous blood samples [30]. Higher variability can bias estimates of the population prevalence anaemia even when mean haemoglobin is unbiased, with the prevalence estimates being more affected when the haemoglobin threshold is further from the population mean. Out of six surveys where the non-DHS survey collected venous blood from NPW and used the same or likely the same HemoCue model, there was no systematic difference in prevalence of anaemia. However, mean haemoglobin in these surveys was near the cutoff for anaemia (ranging from 4 g/l below to 9 g/l above), limiting any effect of possible higher variability in capillary drop haemoglobin.

The differing associations between haemoglobin concentration and type of blood collection in PSC vs NPW in our study may be because collecting capillary blood samples from children is more challenging than collecting samples from women or adults. PSC have smaller fingers and may not wish to provide a blood sample, and thus, “issues” encountered when collecting a capillary sample may compromise the haemoglobin results [31]. Lower haemoglobin concentrations in capillary samples may be attributable to sample dilution from interstitial fluid stemming from finger “milking” [32], shallow lancet punctures which do not yield sufficient blood flow, or cuvettes containing air bubbles: these situations are all more likely to occur when a child is uncooperative. A recent study found poorer correlations between venous and capillary samples in children (1-9 years) compared to adolescents (10-19 years) [27], perhaps indicating that collecting capillary blood samples in younger children was more challenging and more error prone.

Our study did not distinguish results by the blood drop (ie, 2nd vs 4th) used when capillary blood was measured, which have been shown to affect haemoglobin concentrations [16,33,34]. This decision was made because, despite some documentation on blood drop noted in reports, the blood dropped used in the field could not be verified. Our analysis did include at least two non-DHS surveys that measured haemoglobin concentrations from pooled capillary blood (see **Online Supplementary Material**), which has been observed in small studies to contain higher quantities of haemoglobin than single-drop capillary samples [35]. Several studies are currently exploring the extent to which capillary single drop and pooled capillary blood result in different haemoglobin values [36].

### Differences by analytical method

In both PSC and NPW, our study found that mean haemoglobin reported by surveys using HemoCue® Hb 301 devices were consistently higher – by approximately 4 to 6 g/L – than mean haemoglobin reported by surveys using the HemoCue® 201+ device. Other studies have also found differences in mean haemoglobin measured by these devices on the same blood samples from the same individual collected at the same time. When examining the haemoglobin results from multiple comparison studies, Rappaport et al. [8] found that haemoglobin concentrations in venous blood from the HemoCue® Hb 301 were generally higher than an automated haematology analyser used as a reference. Similarly, two studies found that, on average, the haemoglobin measurements from the HemoCue® Hb 301 were 3.4 g/L [16] and 2.6 g/L [15] higher than the HemoCue® Hb 201+. Whitehead et al. [16] also tested the delayed reading of haemoglobin concentrations in both the HemoCue® Hb 201+ and 301 devices, and found a positive association between haemoglobin concentration and increased reading time (ie, time between aspirating the sample into the Hemocuvette and the reading taken on the device) only in the HemoCue® Hb 301 device. While our study did not have data on the time between sample collection and measurement for surveys using the HemoCue® 301, it is plausible that higher haemoglobin concentrations we found in surveys using the 301 device in field conditions were at least partially due to measurements taken after the manufacturer’s recommended time lag (ie, <40 seconds).

### Strengths and Limitations

The key strength of our study is that it compares the results of population-based surveys that were chosen systematically. Comparing results from population-based surveys enabled us to observe the differing haemoglobin concentrations and resulting anaemia prevalences when collected under typical field conditions. Moreover, anaemia prevalences obtained from population-based surveys are frequently used when designing national nutrition policies and programs. Furthermore, the comparison of DHS with non-DHS enabled our study to observe the potential influence of differing methods on national-level haemoglobin and anaemia findings, and the differences between standardized DHS compared to bespoke non-DHS – typically national nutrition surveys.

As the majority of population-representative data on anaemia in highest-burden countries is produced by The DHS Program, our characterization of the DHS as the de facto control was apt. However, an important limitation of our study is the lack of a gold standard comparator to identify the most precise and accurate combination of type of blood collection and analytical approaches used in population-based surveys. In addition, because our design is observational, all effects associated with blood collection type or analytical approach could be caused by some other factor associated with these methodological choices, such as the survey design, lancet type, pooled vs single drop capillary blood collection, or season of data collection. Seasonality, in particular, has been shown to affect the prevalence of anaemia, particularly in malaria-endemic areas where rainy seasons increase the rates malaria transmission [37]. Similar to other studies [9,10], we did not attempt to account for seasonality in the malaria endemic countries included in our study because timing of malaria season varies geographically (including sub-nationally) and from one year to another [38]. Our study was also limited by the relatively small number of near-in-time pairs identified. This was further compounded by our inability to obtain mean haemoglobin and its precision for all included surveys, despite our outreach to survey leads, and resulted in small sample size for comparisons based both on type of blood collection and analytical approach. Our study identified near-in-time pairs from a 21-year time period, which maximized the number of pairs included in the analysis. This long time period introduced a limitation, as older surveys utilized the HemoCue® B, which is no longer available on the market. While the inclusion of from surveys using the HemoCue® B could be considered a limitation, it is noteworthy that the HemoCue® B and 201+ devices use the same reagents and cuvette volume [39,40].

## CONCLUSIONS

Our study adds to the growing body of literature that finds differences in haemoglobin measurements and anaemia prevalence associated with pre-analytic and analytic methods when measuring haemoglobin concentration. Future research is needed to confirm the associations found in our study, so that policy-makers can confidently monitor haemoglobin and anaemia prevalence in populations.

## Additional material


Online Supplementary Document
