# Supplementary material for: Associations between type of blood collection, analytical approach, mean haemoglobin and anaemia prevalence in population-based surveys: A systematic review and meta-analysis
Source: J Glob Health. 2022 Nov 23;12:04088. doi: 10.7189/jogh.12.04088 (PMC9682214; doi:10.7189/jogh.12.04088)
Supplement: Online Supplementary Document [file jogh-12-04088-s001.pdf]

# Online Supplementary Document

# Associations between type of blood collection, analytical approach, mean haemoglobin and anaemia prevalence in population-based surveys: A systematic review and meta-analysis

Authors: Gretchen A Stevens, Monica C Flores-Urrutia, Lisa M Rogers, Christopher J Paciorek, Fabian Rohner, Sorrel Namaste, James P Wirth

## CONTENTS

Appendix S1. Supplementary methods (PRISMA reporting items 5-15) .................................................... 2

Data search, inclusion and access (PRISMA reporting items 5-11)........................................................

2 Initial inclusion of surveys

........................................................................................................... 2 Identification of near-in-time survey pairs ......................................................................................... 3

Inclusion of near-in-time survey pairs ..................................................................................

4 Analysis of individual-level data and outreach to study authors to fill data gaps

.............................. 4

Data items .............................................................................................................................. 5

Table S1 ...............................................................................................................................

6

Risk of bias assessment .......................................................................................................
6

Data analysis (PRISMA reporting items 12-13) ......................................................................... 7

Effect measures .....................................................................................................................

7 Classification of survey pairs

............................................................................................... 8

Table S2 ...............................................................................................................................

9 Descriptive analysis ...............................................................................................................

10 Meta-analysis ........................................................................................................................

11

Meta-regression ................................................................................................................... 11

References ................................................................................................................................. 12

Table S3 ...............................................................................................................................

13 Figure S1

............................................................................................................................ 14

Figure S2 .............................................................................................................................
15

Figure S3 .............................................................................................................................
16

Figure S4 ........................................................................................................................................

17

# Appendix S1. Supplementary methods (PRISMA reporting items 5-15)

## Data search, inclusion and access (PRISMA reporting items 5-11)

Our aim was to identify pairs of population-based studies including haemoglobin measurements that covered the same population at approximately the same time, in order to assess the influence of factors associated with haemoglobin measurement. Therefore, our inclusion criteria were designed to ensure that studies in each pair were comparable in terms of population included and survey timing. This was done by including only data sources that used a probabilistic method to sample from the same general population at approximately the same time. Inclusion of study pairs was done in the following stages:

(1) Initial inclusion of surveys
(2) Identification of all near-in-time survey pairs
(3) Inclusion of near-in-time survey pairs
(4) Data harmonization and outreach to study authors

Each of these are described below and summarized in a flow chart (appendix Figure 1). Data search and inclusion was done separately for preschool-aged children (6-59 months; PSC) and women of reproductive age (15-49 years; WRA).

### Initial inclusion of surveys

WHO maintains a Micronutrients database, part of the Vitamin and Mineral Nutrition Information System (VMNIS), which contains haemoglobin concentrations and anaemia prevalence.[1] Data are identified via periodic searches of bibliographic databases,[1] an international network of collaborators, and periodic consultations with national governments, which uncover data sources not included in bibliographic databases. Survey reports published by national and international agencies are included in this database. Bibliographic database searches are limited to humans, and the following search terms are used:

[1] Bibliographic databases used to identify anaemia data sources: MEDLINE, EMBASE, Web of Science, CINAHL, AGRICOLA, IBECS, SCIELO, LILACS, AIM (AFRO), IMEMR (EMRO), PAHO, WHOLIS, WPRIM (WPRO), IMSEAR, Native Health Research Database

(((national) AND (survey)) OR ((population) AND (prevalence))) AND ((iron status) OR (iron deficiency) OR (anemia) OR (anaemia) OR OR (hemoglobin) OR (haemoglobin) OR (low iron level) OR (transferrin receptor) OR (ferritin) OR (insufficient iron))

---

Studies are included in the World Health Organization (WHO) Micronutrients Database if there is a defined population-based sampling frame; a probabilistic sampling procedure is used and standard, validated data collection techniques and measurement methods and devices were used. There is no language restriction, and if needed, principle investigators are contacted. The verification process for surveys to be validated and published into the VMNIS system consist of review and verification by at least two WHO staff. We accessed this database on 27 May 2021. On July 8, 2021 we also searched for surveys from The Demographic and Health Survey (DHS) Program that were not yet included in the WHO

Micronutrients Database. We used the "Survey Characteristics Search" (https://dhsprogram.com/Methodology/Survey-Search.cfm) with the restriction that the survey should include "Anemia testing", and included all published surveys not yet included in the WHO Micronutrients Database (see appendix Figure 1). From this initial dataset, we excluded the following data:

- Data collected prior to 1995: our interest was in the field performance of HemoCue® devices, which were not widely used in population-based surveys prior to this time
- Data covering populations other than women aged 15-49 years or children aged 6-59 months, because these demographic groups are far more likely to be included in surveys than other groups
- Data collected in a high-altitude country (as defined in a previous study)[2] for which it is not known whether haemoglobin values were adjusted for altitude, as it would not be possible to match on altitude adjustment
- Data collected using a facility-based sampling scheme, because it would not be possible to match on sampling method

Data exclusions were done by one reviewer (GS) using an automated script that evaluated relevant fields of the WHO Micronutrients Database (i.e., any decisions involving reviewer judgment were done when data were initially input into the WHO Micronutrients Database, not when data were excluded for this study).

## Identification of near-in-time survey pairs

To identify near-in-time pairs of surveys, midpoint of fieldwork was computed for each survey by averaging the fieldwork start and end year and month (computed as year + (month-0.5)/12). For four surveys that did not report months of fieldwork, the midpoint was estimated as year + 0.5. Any pairs of surveys in the dataset that were carried out on the same demographic group (PSC or WRA), the same country, and with a difference in fieldwork midpoint less than or equal to 18 months were retained.

## Inclusion of near-in-time survey pairs

Many near-in-time survey pairs were from continuous survey series that share common methods, including survey design, technician training, type of blood collection and analytical approach to measuring blood haemoglobin concentration (e.g., Korea National Health and Nutrition Examination Survey, the Health Survey of England, or continuous DHS). Further, of the global body of anaemia monitoring data, surveys conducted The DHS Program are the leading data source. We included only near-in-time pairs that included one survey conducted by The DHS Program (including both DHS surveys and Malaria Indicator Surveys [MIS], hereafter referred to as DHS) and one independent survey (typically a National Nutrition Survey, hereafter termed non-DHS). This ensured that one survey collected blood by capillary puncture and measured haemoglobin with a HemoCue® device (the DHS) while the non-DHS used a variety of blood collection and analytical methods and/or devices for measuring haemoglobin. Some survey pairs did not cover the same demographic group (i.e., age range, pregnancy status) or geographic area (including urban/rural coverage). In these cases, we reanalyzed survey data to match on these variables to the extent possible (further details on reanalysis below). If it was not possible to match on geographic area or pregnancy status, the survey pair was excluded. All but two survey pairs were matched on age range. The first exception was a study carried out in India which reported summary statistics for all women aged 20-84 years,[3] while the near-in-time DHS did not collect data for women over age 49 years. In India in 2005, an estimated 74% of women aged 20-84 years fell in the age range 20-49 years.[4] The second exception was a nutritional survey carried out in Peru in 2004 which reported statistics for children less than five years of age,[5] presumably including infants 0-5 months of age, an age range which is not subject to haemoglobin measurement in the DHS. The latter exception did not provide statistics on mean haemoglobin. For all surveys in high-altitude countries, we used altitude-adjusted statistics. Smoking-adjusted statistics were used if available, but failure to match on smoking adjustment was not a reason for exclusion, given the small magnitude of the adjustment. Finally, in cases where a survey was conducted within 1.5 years of two other surveys, the closest nearin-time pair was

retained to ensure that data from PSC, non-pregnant women 15-49 years (NPW), and pregnant women 15-49 years (PW) in each survey will only be utilized for a single near-in-time pair.

## Analysis of individual-level data and outreach to study authors to fill data gaps

For some data sources, including all publicly available DHS, de-identified individual-level data from health-examination surveys and household surveys with haemoglobin measurements were available to the authors and were reanalyzed. This allowed for computation of a complete set of summary statistics, including mean haemoglobin, its standard deviation, and effective sample size, following previously established methods.[2]

Some survey methods, including  HemoCue® model, or (in the cases where we could not reanalyze individual-level data) summary statistics were not reported. In these instances, we contacted the principal investigators and researchers affiliated with each survey to request the missing information. Regarding the HemoCue® model used, we also contacted the HemoCue® company and requested the dates when each model was commercially available (personal communication, Lena Wahlhed, Director of Alliance Development, HemoCue®). In brief, the HemoCue® B was the first HemoCue® platform that measured haemoglobin, which was followed in 2002 by the HemoCue® Hb 201+ platforms. The HemoCue® Hb 301 was released in 2006, and the HemoCue® Hb 801 in 2019. Based on this information, we could confidently list the HemoCue® model as the “HemoCue B” for surveys that were conducted prior to 2002.

## Data items

In near-in-time survey pairs, we extracted three primary outcome measures for PSC and for women, separately by pregnancy status if possible:

1) mean haemoglobin concentrations;
2) total anaemia (defined as haemoglobin <110 g/L in PSC and PW, and haemoglobin <120 g/L NPW)
3) severe anaemia (defined as haemoglobin <70 g/L in PSC and PW, and haemoglobin <80 g/L NPW) In order to compute differences in prevalence or mean with uncertainty, it is necessary to have a measure of precision for each survey-statistic pair. These were not consistently reported and were sometimes not available at all. For mean haemoglobin concentration, the measures of precision that that were sought – in order of priority – were design effect, sample size, and standard deviation; standard error; or 95% confidence intervals. For prevalence measures (i.e., all anaemia or severe anaemia), the measures of precision sought – in order of priority – were design effect with sample size, standard error,

and 95% confidence intervals. A full list of survey characteristics extracted from the WHO Micronutrients Database and sought from primary investigators is listed in Table S1.

Table S1. Study characteristics and summary statistics extracted from the WHO Micronutrients Database, computed from individual-level data, and/or requested from primary investigators.

| |
|---|
| Reference information / name of survey |
| Country |
| Geographic representation of the survey (i.e., national, urban or rural, another specific subnational area) |
| Years and months of fieldwork |
| Age range included |
| Pregnancy status (for women) |
| Type of blood collection (i.e., capillary puncture, venipuncture; see Note) |
| Analysis method (i.e., HemoCue®, clinical analyzer) and device model |
| Whether statistics were adjusted for altitude |
| Mean haemoglobin, standard error and confidence interval |
| Standard deviation of haemoglobin |
| Prevalence of total anaemia, as defined by WHO[6], its standard error and confidence interval |
| Prevalence of severe anaemia, as defined by WHO[6], its standard error and confidence interval |
| Sample size |
| Design effect (mean haemoglobin) |
| Design effect (prevalence of anaemia) |
| Design effect (median over multiple statistics, as computed in a previous analysis)[2] |

Note: if known to the authors, pooled vs. single-drop capillary blood collection was noted in the dataset, but this information was not sought from primary investigators.

## Risk of bias assessment

The risk of bias was minimized by excluding studies with high risk of bias. Specifically:

- only surveys that have used a probabilistic sampling method with a defined sampling frame to randomly select participants from the general population were used;
- all surveys in high-altitude countries were adjusted for altitude;
- all surveys pairs that could not be matched on geographic representation and (for women) pregnancy status were excluded;
- surveys were reanalyzed to match on age range to the extent feasible; and
- all survey pairs that could not be matched on anaemia definition were excluded from the relevant analysis.

We reviewed a risk of bias assessment tool developed by Hoy et al. [7] but found that the criteria proposed by the authors were accounted for in our analysis by our exclusion criteria or by population matching. Therefore, it was deemed redundant to carry out further risk of bias assessment.

## Data analysis (PRISMA reporting items 12-13)

All statistical analyses were carried out using Stata version 16.1.[8] Every included survey pair was included in every descriptive/quantitative analysis, provided that the necessary statistics could be obtained. Although we aimed to carry out separate analyses for PW and NPW, some surveys did not report statistics by physiological status. Further, we were able to obtain few survey pairs (8) for PW, and these surveys had small sample sizes, yielding an uninformative dataset. We listed all identified survey pairs with PW data in main text Table 1 but do not include these in main text figures and tables. When we could not obtain data on NPW, we included survey pairs for which data on all WRA were available with survey pairs covering NPW.

### Effect measures

For each survey pair, we computed the following statistics, data permitting:

1) difference in mean haemoglobin concentration;

2) difference in total anaemia prevalence (defined as haemoglobin <110 g/L in PSC and PW, and
<120 g/L in NPW); and

3) difference in severe anaemia prevalence (defined as haemoglobin <70 g/L in PSC and PW, and <80 g/L in NPW).

Mean difference and difference in prevalence (risk difference) and their confidence intervals were computed. Correct computation of the confidence intervals around these differences requires additional statistics from each survey, including effective sample sizes and (for mean difference) and sample standard deviation of haemoglobin. Effective sample size is rarely reported, but is needed to accurately reflect the precision of population statistics derived from complex surveys. We computed or approximated effective sample sizes for all surveys in one of three ways, listed in order of preference:

1. If design effect (DEFF) was reported or could be computed from individual-level data, effective sample size (ESS) was computed as (sample size)/DEFF. If feasible, this was done separately for mean haemoglobin and prevalence of anaemia, since design effects vary by statistic.
2. If the variance of the prevalence of anaemia could be computed from a reported standard error or confidence interval, effective sample size was computed as follows:

$$ESS = \frac{p(1-p)}{\quad}$$

$$var(p)$$

where p is prevalence of anaemia.

3. If data were not available to compute ESS directly, the median design effect (2.1) across 191 population-based surveys was used, following a previously established approach.[2]

Standard deviation of haemoglobin was not available for 6 of 18 non-DHS surveys for which mean haemoglobin was available. None of these surveys reported ESS or DEFF, which precluded computing standard deviation from standard error or confidence intervals of mean haemoglobin. Therefore, median standard deviations of haemoglobin from a database of population-based surveys (15.4 g/L for WRA, and 14.7 g/L for PSC) were used.[2]

The variance of prevalence difference (PD) was estimated using the following formula:[2]

$$Var(PD) = \frac{p(1-p)}{(ESS)} + \frac{p(1-p)}{(ESS)}$$

where the subscript indicates the DHS or non-DHS in the survey pair. The variance of mean difference (D) was computed using the following formula:

$$Var(D) = \frac{s}{ESS} + \frac{s}{ESS}$$

where s is sample standard deviation of haemoglobin.

## Classification of survey pairs

Survey pairs were classified by similarity/differences in type of blood collection and analytical approach to measure haemoglobin concentration. All DHS collected capillary blood. Survey pairs were therefore categorized by the type of blood collected by the non-DHS (capillary or venous).

Table S2 shows the analytical approaches used to measure haemoglobin in the surveys included in our dataset. HemoCue® devices were employed frequently and the chemical approach for measuring haemoglobin varies by device. The HemoCue® B and 201+ analyzers are based on similar methodologies, whereby their cuvettes contain reagents that convert haemoglobin into

[2] Formulas can be found on pages 82-87 of the Stata Meta-Analysis Reference Manual, Release 16.

methaemoglobinazide (HiN3) or azidemethemoglobin, respectively,[9,10] with the absorbance of HiN3 or azidemethemoglobin measured using photometry. In contrast, the cuvettes of the HemoCue® Hb 301 analyzer do not contain reagents, and photometry is used to measure "… the absorbance of whole blood at a Hb/HbO2 isosbestic point" to calculate the haemoglobin concentration.[11] In contrast to HemoCue® devices, laboratory-based methods were rarely used; among the four surveys using

---

laboratory-based methods, several different methods were used (Table S2 and main text Table 1). Therefore, we grouped survey pairs by analytical approach as follows:

- Pairs in which the DHS used a HemoCue® device and the non-DHS used a laboratory-based methods ("Laboratory-based vs. any HemoCue®").
- Pairs in which both surveys were confirmed to have used the same model of HemoCue® device ("Same HemoCue® model").
- Pairs in which both surveys used a HemoCue®, but the model of one survey could not be confirmed. These two survey pairs were conducted in the mid-2000s when most surveys used HemoCue® Hb 201+, thus, we consider that both surveys likely used the same HemoCue® model ("Likely same HemoCue® model"). This category was combined with the previous one for the quantitative analyses.
- Pairs in which both surveys in the pair measured haemoglobin with a HemoCue®, but the model differed; these are identified by the model used in each survey.

Table S2. Details of analytical approaches to measuring blood haemoglobin concentrations used by studies included in the meta-analysis and meta-regression

| Device | Method | Category used for analysis in this study | Surveys in this study using the method and device |
|---|---|---|---|
| HemoCue® B | Photometry of methaemoglobinazide (cuvettes contain reagents that release haemoglobin from red blood cells, which are then converted to methaemoglobinazide) | HemoCue® B | At least 5 DHS and 5 non-DHS surveys listed in main text Table 1 |

| | | | |
|---|---|---|---|
| HemoCue® Hb 201+ | Photometry of azidemethemoglobin (cuvettes contain reagents that release haemoglobin from red blood cells, which are then converted to azidemethemoglobin) | HemoCue® Hb 201+ | At least 14 DHS and 8 non-DHS surveys listed in main text Table 1 |
| HemoCue® Hb 301 | Photometry of whole blood (cuvettes contain no reagents) | HemoCue® Hb 301 | At least 1 DHS and 7 non-DHS surveys listed in main text Table 1 |
| Automated cell counter (COBAX ABX) | Clinical analyzer (chemical based) | Laboratory-based method | National micronutrient survey, Jordan 2002 |
| Photoelectric digital colorimeters. Commercial kits (Dr Reddy's Laboratory, Glaxo Laboratories or Zydus Pathline) were used. | Cyanmethaemoglobin | Laboratory-based method | National Nutrition Monitoring Board survey in rural areas of 9 states, India 2004-2005 |
| Beckman Coulter Cell Counter (Beckman Coulter Inc, 2003) | Clinical analyzer (chemical based) | Laboratory-based method | National micronutrient survey, Jordan 2010 |
| 5 parts automated cell counter (Beckman coulter) | Cyanmethaemoglobin | Laboratory-based method | Comprehensive National Nutrition Survey (CNNS), India 2016-2018 |

## Descriptive analysis

Table 1 (main text) shows all identified pairs and their characteristics, and total anaemia prevalence in each survey is listed in appendix Figure S2. The "public health significance" of anaemia was classified using WHO guidelines [6]. The absolute change in prevalence based on a change in haemoglobin distribution is related to the starting prevalence. For example, a given shift in mean haemoglobin results in a larger change in prevalence if prevalence is near 50% and a smaller change in prevalence if starting prevalence is near 0% or near 100%. Because of this, quantitative meta-analysis of risk difference is not appropriate when underlying prevalence varies, as in our dataset. Therefore, rather than quantitatively

synthesizing data on prevalence of anaemia, the data are displayed graphically (main text Figure 1 and Figures S2 and S3). In addition, we grouped survey pairs by all possible combinations of demography, type of blood collected, and analytical approach. If more than 5 pairs fell into a category, the median risk difference is listed in Table S3; if more than 10 pairs fell into a category, interquartile range is also shown. For mean difference, forest plots were drawn for each population, with survey pairs grouped by type of blood collection (main text Figure 2), analytical approach (main text Figure 3), and by both (Figure S4). Within each group, survey pairs were ordered by year of data collection.

### Meta-analysis

Restricted maximum-likelihood random-effects meta-analyses were carried out. We considered the DHS to be the de facto control group (since all surveys collected capillary blood and used a HemoCue® device) and the non-DHS to be the comparator group. Pre-specified sub-group analyses were carried out on the basis of type of blood collection and analytical approach, classified as described above.

### Meta-regression

Sub-group analyses of difference in mean haemoglobin by type of blood collection and by analytical approach (main paper Figures 2-3) may each be confounded by the other factor. Disaggregation into categories based on both type of blood collection and analytical approach results in small numbers of survey pairs in each category (Figure S4). We performed two restricted maximum-likelihood randomeffects meta-regression, one for each population group, in an attempt to disentangle any effect associated with these factors. Survey pairs were classified as described above. Statistical code is available online at https://osf.io/g895r/ .

## References


1 World Health Organization. Micronutrients database. Geneva; 2021. Available: https://www.who.int/vmnis/database/en/

2 Stevens G, Paciorek C, Flores-Urrutia MC, Borghi E, Namaste S, Wirth JP, et al. National, regional and global estimates of anaemia by severity in women and children for 2000-2019: a pooled analysis of population-representative data. Lancet Glob Heal. 2022;10:E627–39. Available: https://www.thelancet.com/journals/langlo/article/PIIS2214-109X(22)00084-5/fulltext

3 National Institute of Nutrition. Diet and nutritional status of population and prevalence of hypertension among adults in rural areas. Hyderabad; 2006.

4 United Nations Department of Economic and Social Affairs. World Population Prospects 2019. New York: United Nations; 2019. Available: https://population.un.org/wpp/

5 Monitoreo nacional de indicadores nutricionales 2004. Lima; 2004. Available: https://www.ins.gob.pe/insvirtual/BiblioDig/MONIN/M2003/ITM2004.pdf

6 World Health Organization. Haemoglobin concentrations for the diagnosis of anaemia and assessment of severity. Geneva; 2011. Available: https://apps.who.int/iris/handle/10665/85839

7 Hoy D, Brooks P, Woolf A, Blyth F, March L, Bain C, et al. Assessing risk of bias in prevalence studies: modification of an existing tool and evidence of interrater agreement. J Clin Epidemiol. 2012;65:934–9. doi:10.1016/j.jclinepi.2011.11.014

8 StataCorp. Stata: release 16. College Station, TX: StataCorp LLC; 2019.

9 Ickeringill M. The HemoCue B-Haemoglobin Analyzer. In: Update in Anaesthesia. 1999.

10 HemoCue. HemoCue Hb 201+ Operating Manual. Ängelholm, Sweden; 2007.

11 HemoCue AB. Operating Manual - HemoCue® Hb 301. Ängelholm, Sweden; 2020.

Table S3. Difference in total anaemia prevalence in each survey pair, non-DHS less DHS, by type of pair. For each methods comparison category, median prevalence difference is shown if at least 5 survey pairs were identified, and 25th and 75th percentile are shown if at least 10 survey pairs were identified.

| Population | Methods comparison | | Number of survey pairs | Prevalence difference (non-DHS less DHS, in percentage points) | | |
|---|---|---|---|---|---|---|
| | Blood collection (non-DHS)[1] | Analytical approach (non-DHS vs. DHS) | | Median | 25th percentile | 75th percentile |
| Non-pregnant/all women | Capillary | any | 6 | 3.6 | | |
| Non-pregnant/all women | Venous | Same/likely same HemoCue® device | 5 | 0.8 | | |
| Non-pregnant/all women | Venous | any | 10 | -2.4 | -8.5 | 4.6 |
| Non-pregnant/all women | any | HemoCue® Hb 301 vs. HemoCue® Hb 201+ | 5 | -15.3 | | |
| Non-pregnant/all women | any | Same/likely same HemoCue® device | 7 | 1.7 | | |
| Preschool-age children | Capillary | Same/likely same HemoCue® device | 7 | -2.7 | | |
| Preschool-age children | Capillary | any | 10 | -4.8 | -15.7 | 5.3 |
| Preschool-age children | Venous | Same/likely same HemoCue® device | 7 | -11.8 | | |
| Preschool-age children | Venous | any | 13 | -16.9 | -20.3 | -10.3 |
| Preschool-age children | any | HemoCue® Hb 301 vs. HemoCue® Hb 201+ | 6 | -22.2 | | |
| Preschool-age children | any | Same/likely same HemoCue® device | 14 | -7.7 | -11.8 | -2.7 |

Notes

1. All DHS surveys collected blood via capillary puncture.

Figure S1. Data access and inclusion flow chart.

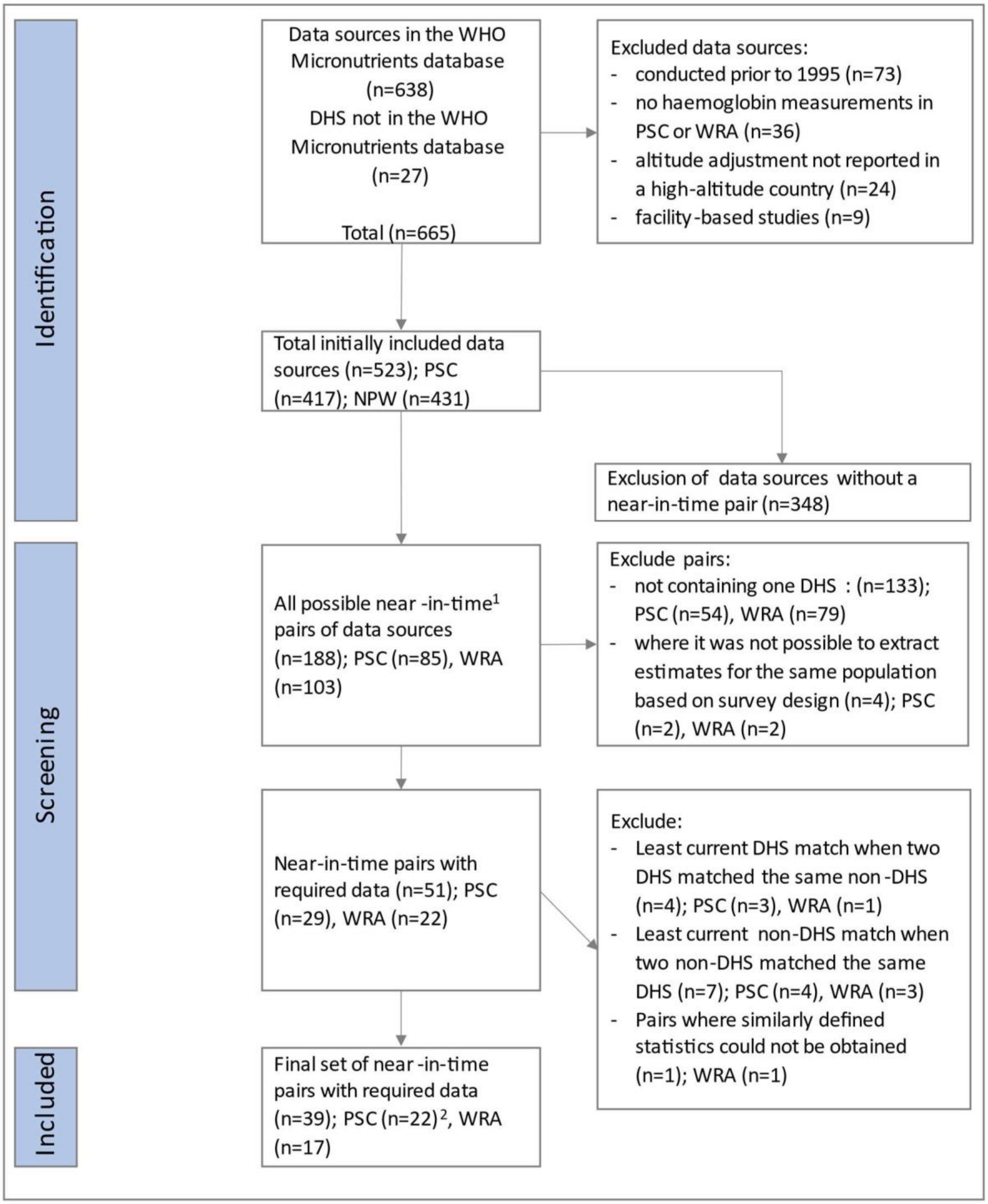


1. Near-in-time pairs = surveys with a fieldwork midpoint within 18 months of each other
2. These 22 survey pairs contribute 23 matched data points because one non-DHS presents data for two geographic areas separately

DHS = Demographic and Health Survey, PSC = preschool aged children, WRA = women of reproductive age

Figure S2. Prevalence of total anaemia in each survey nested within each survey pair, sorted by demographic group and prevalence of total anaemia in the DHS.

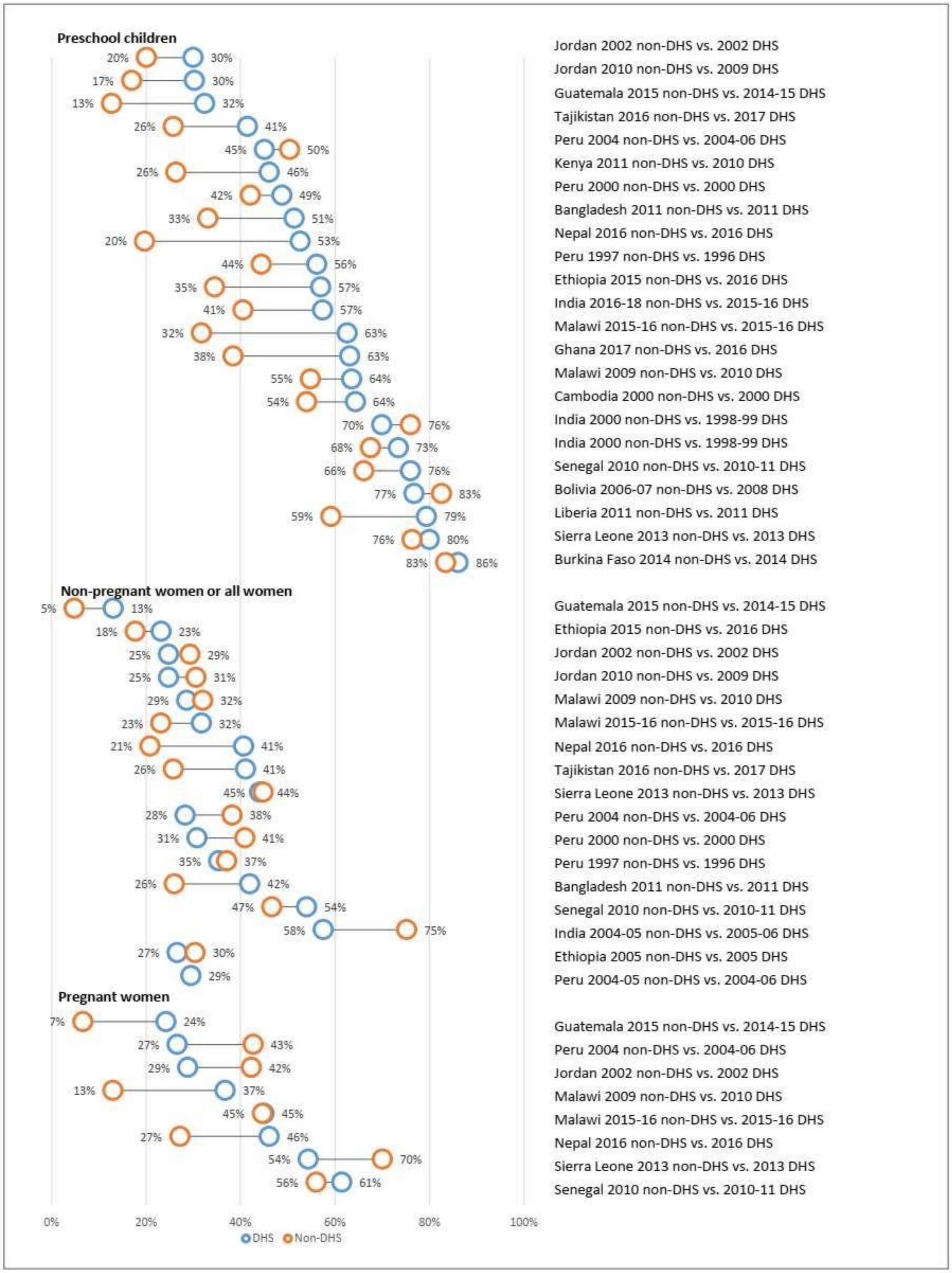

Figure S3. Difference in prevalence of severe anaemia in near-in-time survey pairs, non-DHS less DHS, against prevalence of severe anaemia in the DHS, (A) non-pregnant women/all women, and (B) preschool-aged children.

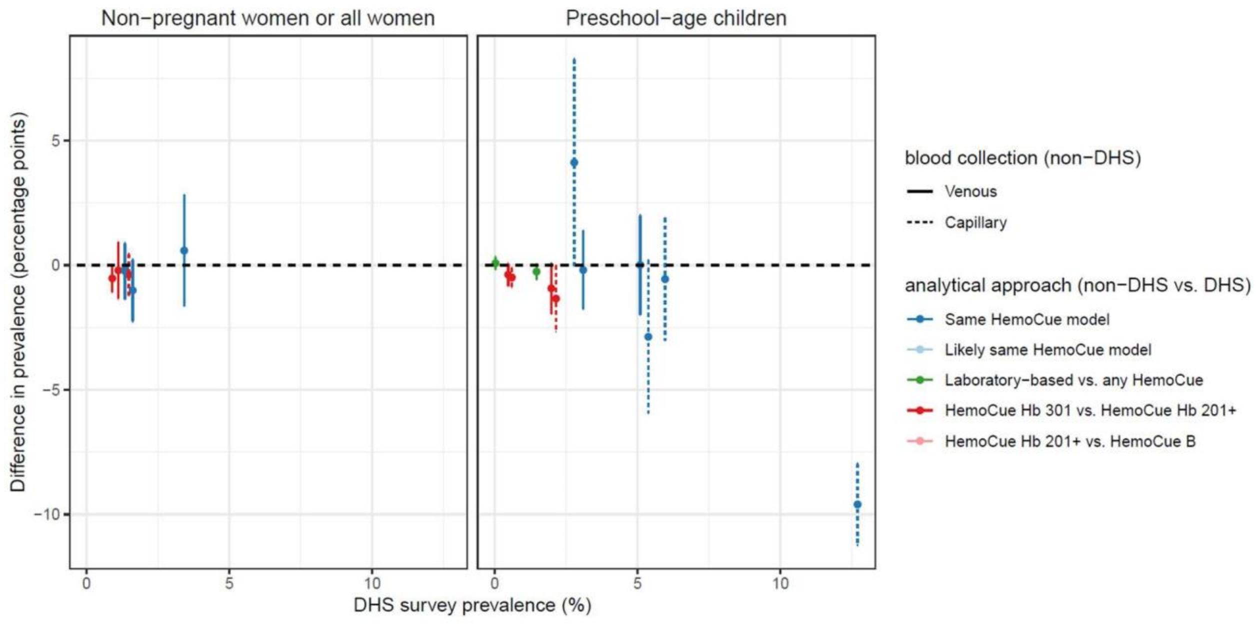


Figure S4. Difference in mean haemoglobin in near-in-time survey pairs, non-DHS less DHS, grouped by type of blood collected in the non-DHS and by concordance/discordance of analytical approach to measure haemoglobin concentration. All DHS collected capillary blood.

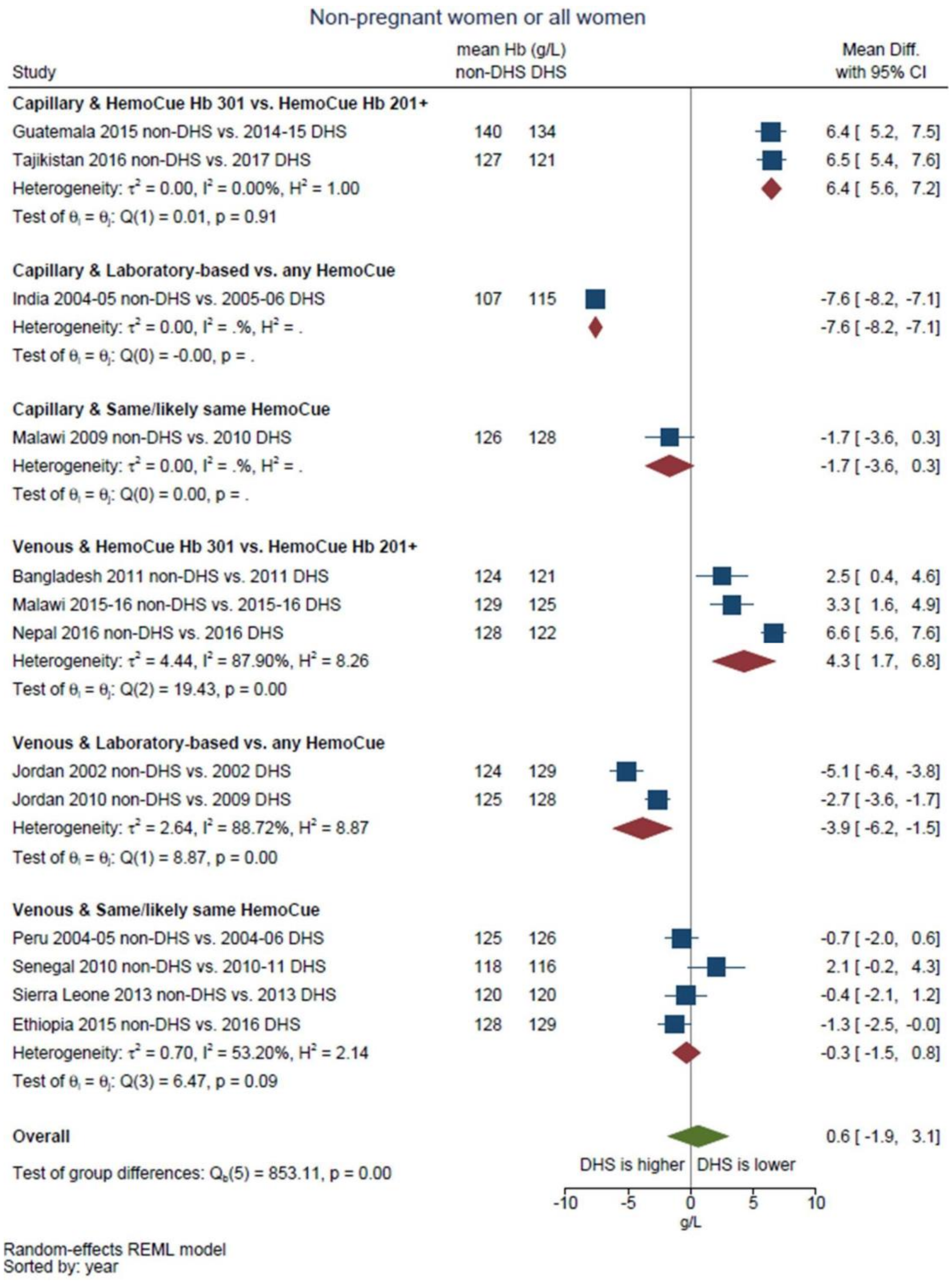


Note: Peru 2004-05 non-DHS and 2004-2006 DHS data include pregnant women. All other data are for nonpregnant women.

Figure S4 (cont). Difference in mean haemoglobin in near-in-time survey pairs, non-DHS less DHS, grouped by type of blood collected in the non-DHS and by concordance/discordance of analytical approach to measure haemoglobin concentration. All DHS collected capillary blood.

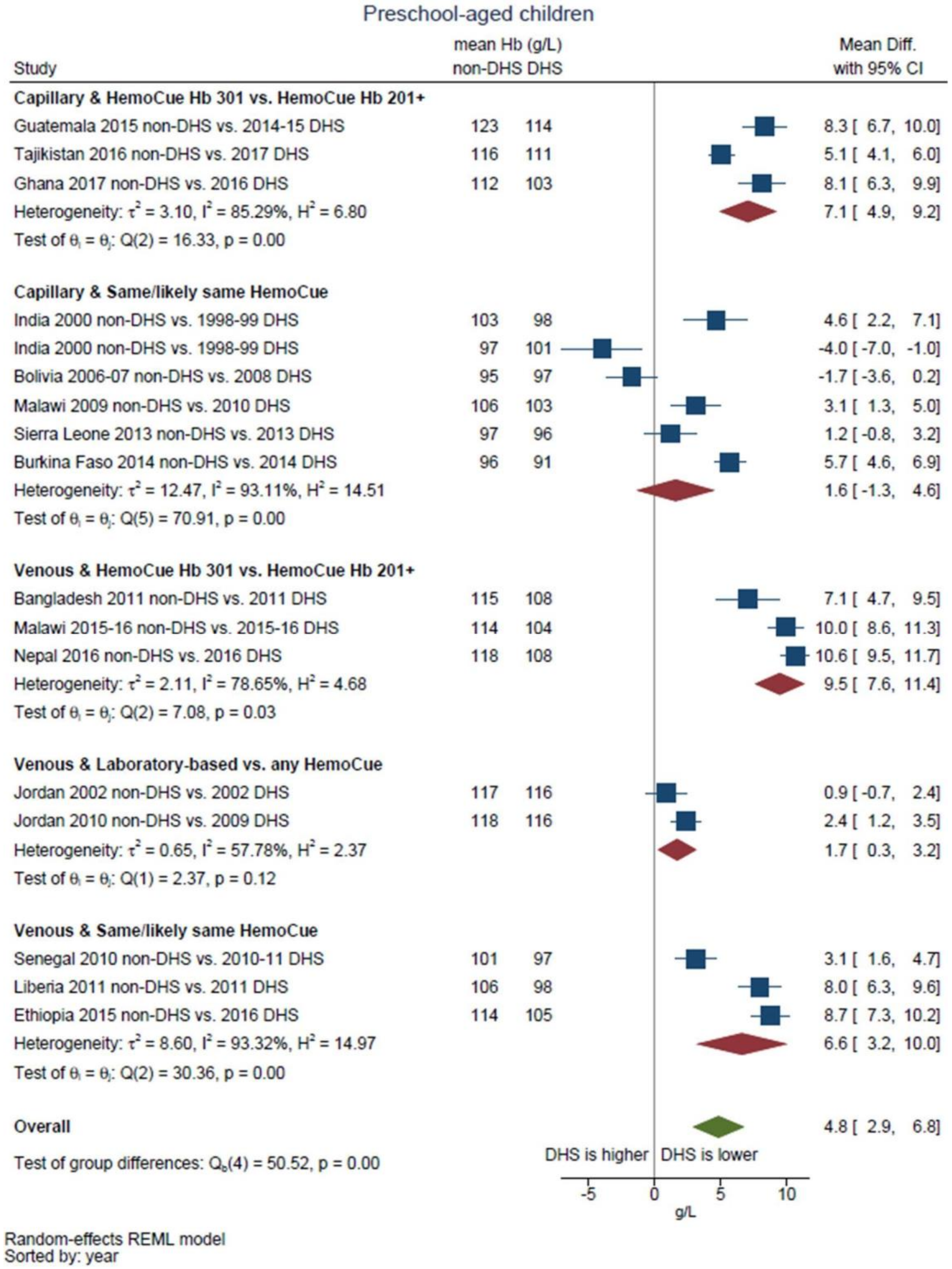


Figure S4 (cont). Difference in mean haemoglobin in near-in-time survey pairs, non-DHS less DHS, grouped by type of blood collected in the non-DHS and by concordance/discordance of analytical approach to measure haemoglobin concentration. All DHS collected capillary blood.

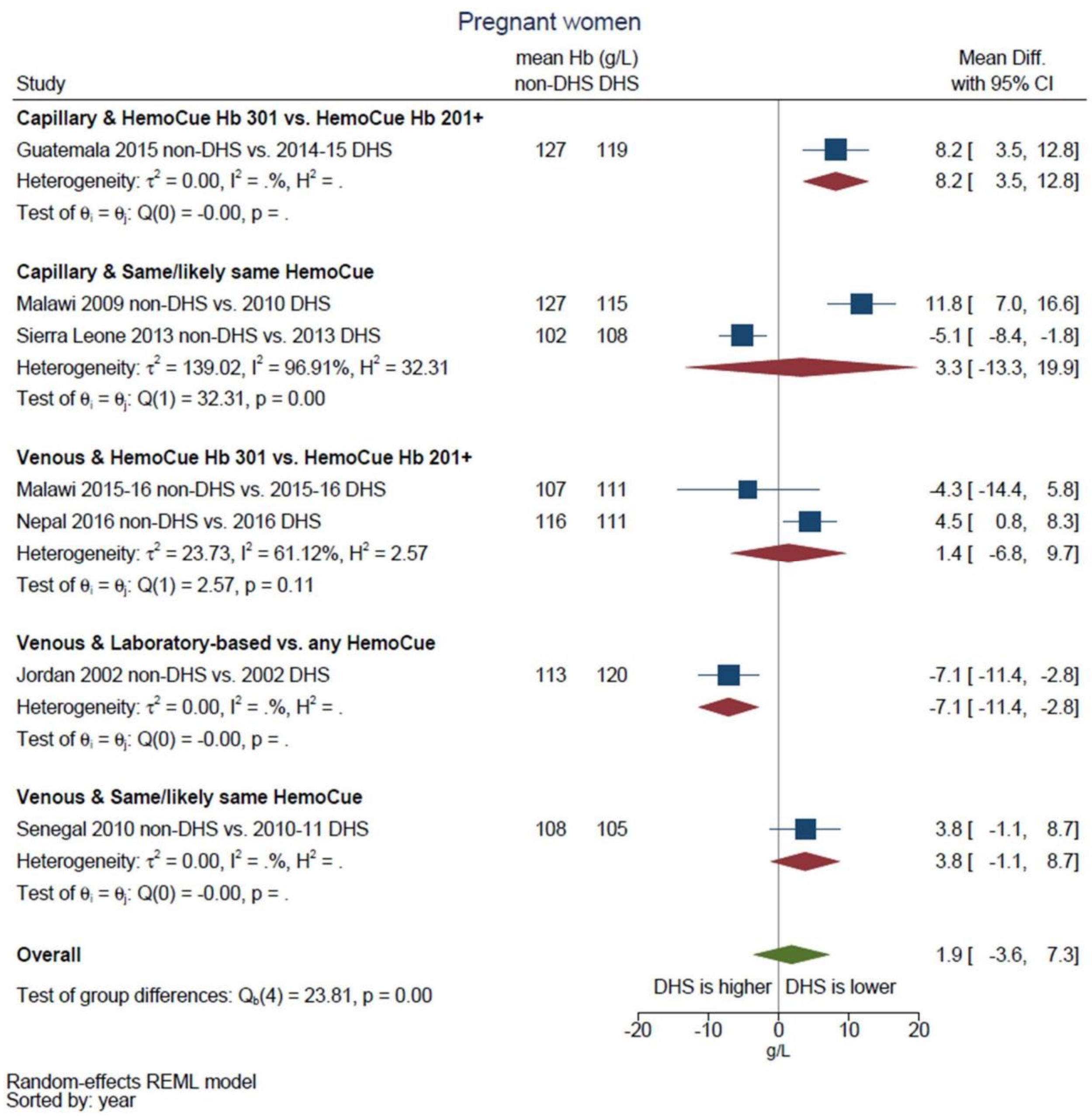
Pregnant women
Study
mean Hb (g/L)
non-DHS DHS
Mean Diff.
with 95% CI
Capillary & HemoCue Hb 301 vs. HemoCue Hb 201+
Guatemala 2015 non-DHS vs. 2014-15 DHS 127 119 8.2 [ 3.5, 12.8]
Heterogeneity: τ² = 0.00, I² = .%, H² = . 8.2 [ 3.5, 12.8]
Test of θi = θj: Q(0) = -0.00, p = .
Capillary & Same/likely same HemoCue
Malawi 2009 non-DHS vs. 2010 DHS 127 115 11.8 [ 7.0, 16.6]
Sierra Leone 2013 non-DHS vs. 2013 DHS 102 108 -5.1 [ -8.4, -1.8]
Heterogeneity: τ² = 139.02, I² = 96.91%, H² = 32.31 3.3 [ -13.3, 19.9]
Test of θi = θj: Q(1) = 32.31, p = 0.00
Venous & HemoCue Hb 301 vs. HemoCue Hb 201+
Malawi 2015-16 non-DHS vs. 2015-16 DHS 107 111 -4.3 [ -14.4, 5.8]
Nepal 2016 non-DHS vs. 2016 DHS 116 111 4.5 [ 0.8, 8.3]
Heterogeneity: τ² = 23.73, I² = 61.12%, H² = 2.57 1.4 [ -6.8, 9.7]
Test of θi = θj: Q(1) = 2.57, p = 0.11
Venous & Laboratory-based vs. any HemoCue
Jordan 2002 non-DHS vs. 2002 DHS 113 120 -7.1 [ -11.4, -2.8]
Heterogeneity: τ² = 0.00, I² = .%, H² = . -7.1 [ -11.4, -2.8]
Test of θi = θj: Q(0) = -0.00, p = .
Venous & Same/likely same HemoCue
Senegal 2010 non-DHS vs. 2010-11 DHS 108 105 3.8 [ -1.1, 8.7]
Heterogeneity: τ² = 0.00, I² = .%, H² = . 3.8 [ -1.1, 8.7]
Test of θi = θj: Q(0) = -0.00, p = .
Overall 1.9 [ -3.6, 7.3]
Test of group differences: Qb(4) = 23.81, p = 0.00
DHS is higher
DHS is lower
-20 -10 0 10 20
g/L
Random-effects REML model
Sorted by: year
